# Dynamic Neural State Identification in Deep Brain Local Field Potentials of Neuropathic Pain

**DOI:** 10.3389/fnins.2018.00237

**Published:** 2018-04-11

**Authors:** Huichun Luo, Yongzhi Huang, Xueying Du, Yunpeng Zhang, Alexander L. Green, Tipu Z. Aziz, Shouyan Wang

**Affiliations:** ^1^Suzhou Institute of Biomedical Engineering and Technology, Chinese Academy of Sciences, Suzhou, China; ^2^University of Science and Technology of China, Hefei, China; ^3^Neural and Intelligence Engineering Center, Institute of Science and Technology for Brain-Inspired Intelligence, Fudan University, Shanghai, China; ^4^Nuffield Department of Surgical Sciences, University of Oxford, Oxford, United Kingdom

**Keywords:** neural oscillation, neural state, local field potential, synchronization, adaptive deep brain stimulation

## Abstract

In neuropathic pain, the neurophysiological and neuropathological function of the ventro-posterolateral nucleus of the thalamus (VPL) and the periventricular gray/periaqueductal gray area (PVAG) involves multiple frequency oscillations. Moreover, oscillations related to pain perception and modulation change dynamically over time. Fluctuations in these neural oscillations reflect the dynamic neural states of the nucleus. In this study, an approach to classifying the synchronization level was developed to dynamically identify the neural states. An oscillation extraction model based on windowed wavelet packet transform was designed to characterize the activity level of oscillations. The wavelet packet coefficients sparsely represented the activity level of theta and alpha oscillations in local field potentials (LFPs). Then, a state discrimination model was designed to calculate an adaptive threshold to determine the activity level of oscillations. Finally, the neural state was represented by the activity levels of both theta and alpha oscillations. The relationship between neural states and pain relief was further evaluated. The performance of the state identification approach achieved sensitivity and specificity beyond 80% in simulation signals. Neural states of the PVAG and VPL were dynamically identified from LFPs of neuropathic pain patients. The occurrence of neural states based on theta and alpha oscillations were correlated to the degree of pain relief by deep brain stimulation. In the PVAG LFPs, the occurrence of the state with high activity levels of theta oscillations independent of alpha and the state with low-level alpha and high-level theta oscillations were significantly correlated with pain relief by deep brain stimulation. This study provides a reliable approach to identifying the dynamic neural states in LFPs with a low signal-to-noise ratio by using sparse representation based on wavelet packet transform. Furthermore, it may advance closed-loop deep brain stimulation based on neural states integrating multiple neural oscillations.

## Introduction

Deep brain local field potentials (LFPs) contain rich information regarding the function of subcortical nuclei in humans (Friston et al., 2015). LFPs exhibit oscillatory behaviors in different frequency bands. Such neural oscillations are simultaneously involved in the neurophysiological and neuropathological functions of nuclei in conditions such as neuropathic pain (Ploner et al., 2017), Parkinson's disease (Hammond et al., 2007; Oswal et al., 2013; Brittain and Brown, 2014), and dystonia (Neumann et al., 2012; Whitmer et al., 2013).

In patients with neuropathic pain, the levels of pain and pain modulation are correlated with the power of oscillations in LFPs, such as theta, alpha, and beta oscillations. LFPs from the ventro-posterolateral nucleus of the thalamus (VPL) in neuropathic pain exhibit increased power of 17–30 Hz oscillations when pain intensity increases (Green et al., 2009). Similarly, the activities of 6–9 and 22–33 Hz oscillations are significantly related to the effects of deep brain stimulation (DBS) for the treatment of neuropathic pain (Huang et al., 2016b). By contrast, the periventricular gray/periaqueductal gray (PVAG) LFPs exhibit increased power of 8–12 Hz oscillations when pain intensity increases (Green et al., 2009). The activities of both 6–9 and 10–12 Hz oscillations are significantly related to the DBS treatment effect for neuropathic pain (Huang et al., 2016b). Subsystems may operate at different frequencies and can form local oscillatory networks in given nuclei (Priori et al., 2004).

In addition to the specific frequency bands, the dynamics of neural oscillations are also related to neuropathic pain. We previously found that the occurrence of 8–10 Hz spindle-shaped oscillations is correlated with pain intensity (Green et al., 2009). The spindle-shaped oscillations can be characterized as synchronization oscillations with higher power and more regular patterns, which may arise from synchronization of neuronal assemblies, i.e., the synchronization of activity within groups of neurons (Buzsaki, 2004; Sarnthein and von Stein, 2009; Buzsáki et al., 2012). The emergence and disappearance of these synchronization oscillations can be thought of as a type of “neural state” of the nucleus. Our previous studies have demonstrated that neural activity features combining theta, alpha, beta, and gamma oscillations are related to pain modulation (Huang et al., 2016b, 2018). The dynamic identification of these neural states would be useful in representing complex brain functions and developing state-specific neuromodulation approaches. Pain modulation may be more specifically related to neural states in the high-dimensional feature space of amplitude, balancing and coupling measures (Huang et al., 2016a), and such neural states may be useful in representing complex brain functions. Dynamic identification of neural states may also be beneficial for studying brain functions in patients with neurological or psychiatric diseases (Neumann et al., 2014). However, there are three challenges to dynamically identifying the neural state: (1) enhancing the representation of the synchronization features in LFPs containing rich information, (2) adaptive discrimination of the synchronization level in non-stationary LFPs, and (3) reliable identification of the neural state in LFPs with a high level of background noise. LFPs can be modeled as $f\left(t\right)=\hat{f}\left(t\right)+\varepsilon \left(t\right)$, which represents the dynamically synchronizing oscillations $\hat{f}\left(t\right)$ that are of interest but hidden in high levels of background noise, ε(*t*). $\hat{f}\left(t\right)$ can be characterized by a sparse representation that has been used for neural signal processing (Wen et al., 2016; Miao et al., 2017). The wavelet packet (WP) transform provides an efficient way to characterize the dynamic change of $\hat{f}\left(t\right)\;$(Donoho and Johnstone, 1994). It enhances the oscillation representation compared to noise and captures the oscillation of interest using a selective wavelet basis. Moreover, the thresholding model in wavelet transformation provides a statistically optimized estimation of $\hat{f}\left(t\right)$ (Donoho and David, 1995).

The two issues to overcome in identifying synchronization of neural oscillations are on-line processing and the influence of high-level noise. First, there is a trade-off between time delay and the accuracy of state identification in online processing. Having fewer data leads to quicker real-time processing but less-accurate state identification. Second, the noise is greater than the synchronization oscillations, and most signal processing approaches are sensitive to noise. In this study, a three-stage hierarchical approach was developed by enhanced representation of the neural oscillations with wavelet packet transform, adaptive thresholding for synchronization level discrimination and binary coding for neural state encoding. The current study aimed to dynamically identify the neural states of nuclei based on the sparse representation of the multiple neural oscillations of the LFPs via a windowed WP transform. Oscillations were sparsely represented in the WP domain, and their dynamic synchronization states were discriminated by comparing WP coefficients of oscillations to adaptive thresholds. Finally, the synchronization state was coded by a binary coding method. Then, the neural states of the PVAG and VPL were derived from the binary coding by using synchronization states of theta and alpha neural oscillations separately or together.

There have been several attempts at automatic detection of neural oscillations, and the approaches mainly involve oscillation extraction and state detection (Staba et al., 2002; Le Van Quyen and Bragin, 2007). Both a band-pass filter and a wavelet transform have been used for oscillation extraction (Wallant et al., 2016). The wavelet transform provides an efficient way to characterize the dynamic changing of neural signals as a commonly used spare representation method (Wen et al., 2016). However, most approaches have been used in off-line processing, and the influence of the short window and level of noise on sensitivity and specificity has been less well-investigated.

## Materials and methods

### Materials

#### Subjects

Sixteen patients with neuropathic pain were recruited (age, 47.3 ± 11.3 years; mean ± *SD*). Ten patients underwent unilateral implantation of deep brain stimulation electrodes in both the VPL and the PVAG, one patient underwent bilateral implantation in the PVAG, and the remaining five patients underwent unilateral implantation in the VPL or the PVAG. All deep brain stimulation implantations were performed at the John Radcliffe Hospital, Oxford. The study was approved by the Oxford Local Ethics Committee (OxRec B), and informed written consent was provided by all patients.

#### Deep brain LFP recording

The surgical procedures for targeting and implantation of DBS electrodes (models 3387™, Medtronic®) have been previously reported (Bittar et al., 2005; Green et al., 2006; Owen et al., 2006b). The DBS target structures were localized on the fused CT/MRI images using Radionics Image Fusion™ and Stereoplan™ (Radionics, MS, USA) pre-operatively. Electrode implantation was then performed under local anesthesia. The final electrode placement and localization of each electrode contact were confirmed for all patients using post-operative stereotactic MRI or CT with fusion to the pre-operative MRI.

The LFPs were recorded from the VPL and/or the PVAG post-operatively via the externalized DBS electrodes. There were 12 recordings from the VPL (either side) and 15 recordings from the PVAG. The LFPs were recorded while the patients were off medication and before the stimulation was turned on for trial stimulation or after the stimulation was turned off overnight. Bipolar LFPs were recorded from three adjacent pairs of deep brain electrode contacts (contacts 0–1, 1–2, and 2–3) with a common electrode placed on the surface of the mastoid. The recordings were made when patients were seated at rest, and any artifacts were carefully identified and excluded. The LFPs were amplified using an isolated CED 1902 amplifier (×10,000, Cambridge Electronic Design, Cambridge, UK), filtered between 0.5 and 500 Hz, digitized using a CED 1401 Mark II at a sampling rate of 2000 Hz, displayed on-line and saved onto a hard disk in Spike2 (Cambridge Electronic Design, UK).

#### Pain assessment

All patients were asked to rate their pain on a visual analog scale (VAS, 0–10, 0 = no pain, 10 = worst pain ever experienced) in a pain diary twice daily (am and pm) over a period of 7 consecutive days. The assessment was performed both before and after DBS surgery (Owen et al., 2006a; Pereira et al., 2010; Boccard et al., 2013). VAS scores of pain assessment between 6 and 12 months after surgery were used to quantify the pain relief as a result of deep brain stimulation. The 14 VAS scores over 7 days were averaged to yield the average pain scores of the pre-operative and post-operative stages. Pain relief by DBS was computed as the post-operative percentage change in the VAS score against the pre-operative score for each patient. The clinical information for all patients is provided in Table 1.

**Table 1:** Demographics, diagnosis, stimulation parameters, and pain assessment of patients.

**Case**	**Age/Sex**	**Diagnosis**	**Targets**	**Stimulation parameters**			**VAS**		
				**A (V)**	**F (Hz)**	**PW (ms)**	**Pre-op**	**Post-op**	**Relief (%)**
1	60/M	Poststroke pain	PAG	0.5	20	360	9.2	8.2	11
			VPL	2	20	450			
2	39/M	Trigeminal neuralgia	PAG	3	20	330	7.5	6	20
			VPL	3.4	20	330			
3	40/F	Intractable forehead pain	PVG	4.8	20	240	5	3	40
			VPL	1.7	20	180			
4	38/M	Phantom limb pain	PAG	2.5	30	120	8	6	25
			VPL	2.4	30	120			
5	43/M	Phantom limb pain	PVG	–	–	–	5.6	2	64
			VPL	4	25	60			
6	53/M	Brachial plexus injury	PVG	2.1	20	240	4.8	4.5	6
			VPL	0.7	20	270			
7	54/M	Poststroke pain	PVG	1.2	10	270	6.7	5.1	24
			VPL	2	10	180			
8	58/M	Facial pain	PVG	3	40	120	9	7.5	17
			VPL	0.8	40	120			
9	42/M	Radiculo-plexopathy	PVG	–	–	–	10	8	20
			VPL	–	–	–			
10	35/M	Cephalalgia	PVG	1.8	50	330	9	8.5	5.56
			VPL	2.8	90	240			
11	58/M	Amputation (phantom limb)	PVG	1.5	7	120	7	6.2	11.4
12	57/F	Amputation (phantom limb)	PAG	4.5	30	450	6.4	6	6.25
			PAG	3.5	30	450	6.4	6	6.25
13	31/M	Radiculo-plexopathy	PVG	–	–	–	6.7	3	55.2
14	33/M	Cephalalgia	PAG	2.8	20	450	6.4	2	68.7
15	46/M	Stroke	VPL	1	15	300	9.8	3	69.4
16	69/M	Brachial Plexus	VPL	–	–	–	8.3	8	3.6

*The implant places of DBS electrodes (Targets) were given. Then, the electric stimulation parameters for treatment are the voltage (A), frequency (F), and power width (PW).The VAS score before operation (pre-op) and after operation (post-op) are given. Then, the pain relief of DBS was calculated using VAS scores before and after DBS surgery*.

#### Pre-processing

The signals were first pre-processed. Segments of 50 s from each LFP recording were selected for further analysis. Because the LFP oscillatory activity energy is mainly concentrated in the frequency band below 90 Hz, the selected LFPs were low-pass filtered at 90 Hz with a tenth-order Chebyshev Type I filter. All LFPs were then down-sampled to 500 Hz. An adaptive notch filter was applied to remove 50 Hz interference, and the LFPs were high-pass filtered at 2 Hz with a fifth-order Chebyshev Type I filter to eliminate baseline shifting. Finally, the LFPs were resampled to 384 Hz so that the theta and alpha oscillations were situated at the [6 3] and [6 2] nodes of the wavelet packet tree. The frequency ranges of nodes [6 3] and [6 2] were 6–9 and 9–12 Hz, respectively.

#### Simulation signal

The simulation signal was generated according to four types of oscillatory characteristics of LFP (Huang et al., 2016b), i.e., frequency, signal-to-noise ratio (SNR), temporal changes and duration. The simulation signal consisted of a sinusoidal signal, a trapezoidal envelope and white noise. First, the frequency of the sinusoidal signal was the central frequency of the theta (7.5 Hz) or alpha (10.5 Hz) oscillations, and its value ranged from −1 to 1. Second, to simulate the temporal change of oscillations, a trapezoidal envelope was generated and multiplied by the sinusoidal signal. The period of the trapezoidal envelope was 2 s, its duty cycle was 0.5, and its amplitude range was 0 to 1. Finally, a certain amount of Gaussian white noise was added to the amplitude-modulated signal to obtain a simulated oscillation signal with a certain SNR. To evaluate the SNR of LFPs for specific oscillations, the theta or alpha oscillations and the background noise were separated by thresholding with the WP transform. A total of 216 segments of 6 s from 27 LFP recordings were used for the calculation. The results are listed in Table 2. The mean SNR of theta oscillations was −9 ± 5.0 dB, and the mean SNR of alpha oscillations was −11 ± 5.5 dB.

**Table 2:** The evaluated signal-to-noise ratio (SNR) of theta and alpha oscillations in PVAG and VPL LFPs.

**Oscillations**	**Mean SNR (dB)**	**Variance (dB)**
Theta (PVAG)	−9.01	4.99
Theta (VPL)	−8.98	3.55
Alpha (PVAG)	−11.12	5.38
Alpha (VPL)	−11.12	5.49

### Methods

**Figure 1:**
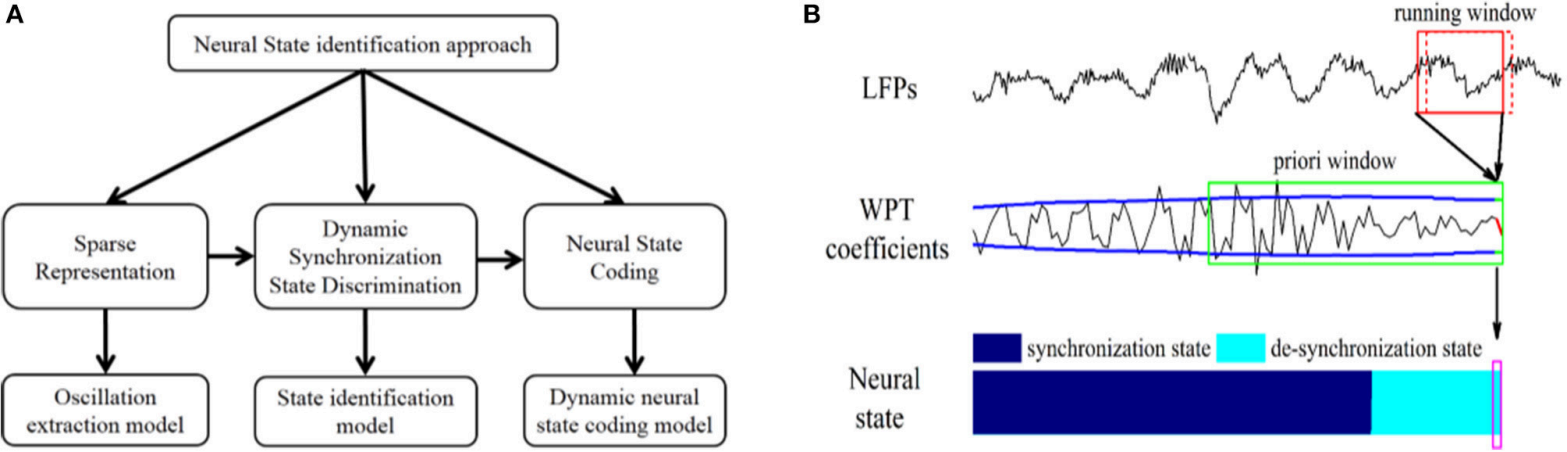
**(A)** Composition of the neural state identification approach. **(B)** Neural state identification. The WP transform is performed with a running window (the red solid box in the first row) on LFPs to calculate the latest WP coefficients. Then, the a priori window (the green solid box at the second row) is updated with these WP coefficients (red line in the second row) to calculate the latest threshold (green lines in the second row). The latest neural state (shown in the purple box) is identified by comparing the latest WP coefficients with the latest threshold synchronization state. For each identification, the running window slides forward in 20 ms steps (the red dashed box at the first row).

The state identification approach was developed to dynamically identify the neural state of the VPL and the PVAG corresponding to neuropathic pain by dynamically discriminating the synchronization state of theta and alpha oscillations. As shown in Figure 1A, the state identification approach includes three models. In the oscillation extraction model, the WP transform was applied to LFPs, and the theta and alpha oscillations were sparsely represented in the WP domain. Then, an adaptive threshold was estimated for discriminating the synchronization state of theta and alpha oscillations in the state discrimination model. Finally, the neural state of neuropathic pain was represented by the synchronization states of the theta and alpha oscillations using a binary encoding method, and the relationship between neural states and pain relief was assessed.

#### Oscillation extraction model

The oscillation extraction model was constructed based on the WP transform (for more information about the wavelet packet transform, please see supplementary material) (Percival and Walden, 2006), and the amplitude of WP coefficients represented the synchronization degree (Figure 1B). To better represent and extract specific oscillations, the WP basis and running window length were optimized, and the performance was validated according to entropy and energy.

##### Optimal WP basis selection

To more effectively extract oscillations, the WP bases were selected for theta and alpha oscillations of the VPL and PVAG LFPs. The VPL and PVAG LFPs exhibited specific synchronization oscillations, which showed patterns with higher regularity and periodicity. The neural oscillations are better represented if the WP basis has similar patterns to the neural oscillations, and the entropy of the WP coefficients has lower value in such a condition. Entropy has previously been used as a criterion for optimal WP basis selection (Samar et al., 1999). There were three steps:

Step 1: General principles. A group of WP bases was chosen according to characteristics of the wavelet basis function, i.e., smoothness of the shape, compacted support, orthogonality, symmetry, and high-order vanishing moment (Banjanin et al., 2001; Peterson et al., 2015).
Step 2: Special principles. The WP coefficients of the theta and alpha oscillations were obtained with the WP transform using the chosen WP basis from step 1. Then, the entropy of WP coefficients was calculated for each different basis. The entropy of WP coefficients u = {u(*k*)} is defined by:
  $$H(u)=\sum_{k}u(k)log(1/u(k))$$
  where *k* is the number of WP coefficients over time.
Step 3: Compare the entropy of the WP coefficient for each WP basis. The lowest entropy indicated the most suitable WP basis among the selected group of bases.

##### Optimal signal running window length

The neural oscillations were dynamically captured with a running window over the LFPs, i.e., the wavelet packet transform was consecutively performed within a short window with 20 ms overlap (Figure 1B). The length of the running window (L_running_) was optimized by evaluating the signal representation abilities of running windows of different lengths. To evaluate the representation ability of a running window of a certain length, two types of signals were generated. The first type of signal x(*k*) was obtained by reconstructing the WP coefficients of one node on the WP tree after performing the WP transform on a 6-s LFP signal without a running window. The second type of signal y(*k*) was obtained by reconstructing WP coefficients of the same node on the WP tree after performing the WP transform on the same 6-s LFP signal with a certain length running window. Consider x(*k*) as the reference signal and y(*k*) as the experimental signal. A relative error (Zalay et al., 2009) between x(*k*) and y(*k*) was the representation ability of this running window. The relative error was designated as the percentage root mean squared deviation between the experimental signal y(*k*) and reference signal x(*k*), which was normalized by the mean value of the reference signal, $\hat{x}$:

$$e=\frac{1}{\hat{x}}{\left[\frac{1}{N}\sum_{k=1}^{N}{\left(y(k)-x(k)\right)}^{\wedge }\;2\right]}^{½}\cdot 100\%$$

In this study, a total of 96 6-s LFP segments from the PVAG and 120 segments from the VPL were used. The WP transform of LFPs was performed with different length running windows, and the experimental signals were the theta and alpha oscillations reconstructed from all of the WP coefficients at nodes [6 3] and [6 2], respectively. The reference signals were the oscillations reconstructed from the WP coefficients at nodes [6 3] and [6 2] obtained after the WP transform without a running window. Then, the experimental signals were compared to the reference signals to calculate the relative error between them. The performance was evaluated running windows of lengths ranging from 32 to 200 points.

For the real application, to perform a real-time calculation, the moving step of the running window was 20 ms.

#### State discrimination model

In the state discrimination model, the synchronization state of each neural oscillation was discriminated using an adaptive threshold. The adaptive threshold was calculated with the WP coefficients from the a priori window, and the WP coefficients from the current running window were compared to the threshold to determine its synchronization state (Figure 1B).

##### Threshold estimation

The “minimaxi” method was used to estimate the threshold due to its advantages of lower estimation variance and greater robustness to noise for short data segments (Tikkanen, 1999; Vidakovic, 1999). The threshold *T* is defined as follows:

$$T=\sigma (0.396+0.1829\cdot \log_{2}N)$$

where σ is the standard deviation of noise and N is the number of WP coefficients. σ was estimated from the median of the WP coefficients u(*k*):

σ≈1/0.6745·Med(|u(k)|)

where *Med*(|u(*k*)|) is the median value of the data sequence {|u(*k*)|}.

##### Optimal length of the a priori window

An a priori window over the WP coefficients was used to calculate the adaptive threshold for state discrimination in the current running window. The length of the a priori window (L_priori_) affects the reliability of estimation of the adaptive threshold. A longer window length leads to less adaptation to temporal changes of the signal, and a shorter window length leads to less robustness and accuracy of the estimation.

The optimal a priori window length was selected by achieving a high sensitivity and specificity of state identification with simulation signals. In this experiment, five different L_priori_ window lengths, 1, 2, 4, 6, and 8 s, were compared. The state was classified as synchronization if at least one of the WP coefficients was beyond the threshold. By contrast, the state was classified as de-synchronization if there was no coefficient beyond the threshold.

The sensitivity and specificity were calculated as:

$$\begin{array}{l} Sensitivity=t_{s}/T_{s}\cdot 100\%\\ Specificity=t_{d}/T_{d}\cdot 100\% \end{array}$$

where *T*_*s*_ and *T*_*d*_ are the timings of true synchronization and de-synchronization states, respectively, and *t*_*s*_ and *t*_*d*_ are the timings of identified synchronization and de-synchronization states, respectively. The true synchronization state was defined as an amplitude of simulated oscillations greater than 0.5, and the de-synchronization state was defined as an amplitude less than 0.5.

The a priori window was updated every 20 ms with the moving step of the running window.

##### Optimal discrimination strategy

The synchronization state of the signal within the current running window was discriminated by comparing the WP coefficients with the adaptive threshold. To improve the sensitivity and specificity, the discriminant strategy was further optimized using additional parameters. The state was classified as “synchronization” if there was at least one WP coefficient in the current running window beyond the threshold, and similar occurrences were observed for the next *n1* consecutive running windows. Otherwise, the state of the current window was considered to be the same as that of the previous window. By contrast, the state was classified as “de-synchronization” if there were no coefficients beyond the threshold and similar occurrences observed for the next *n2* consecutive running windows. Otherwise, the state of the current window was considered to be the same as that of the previous window. The parameters *n1* and *n2* were designed to reduce noise interference, thereby improving the discrimination accuracy.

The parameters *n*_1_ and *n*_2_, each ranging from 1 to 6, were compared, and the sensitivity and specificity were calculated to evaluate the discrimination performance based on each simulated signal.

#### Dynamic neural state for pain

The neural states related to neuropathic pain can be represented by one or more neural oscillations. In this study, binary encoding was used to encode the synchronization. A value of 1 indicated the synchronization state of the neural oscillations, and a value of 0 indicated the de-synchronization state. The symbols α^0^, α^1^, θ^0^, and θ^1^ represent the alpha and theta oscillations of the de-synchronization and synchronization states, respectively. If the states were defined according to both theta and alpha oscillations, there were four states, i.e., α^0^θ^0^, α^0^θ^1^, α^1^θ^0^, and α^1^θ^1^.

In this study, theta and alpha oscillations were both used to encode the states related to neuropathic pain. Therefore, there were a total of six states: α^1^, θ^1^, α^0^θ^0^, α^0^θ^1^, α^1^θ^0^, and α^1^θ^1^. The relationships between the occurrence frequencies of these six states, the pain level before surgery and the pain relief by DBS were quantified by Spearman correlation. The occurrence frequency was defined as the percentage of the occurrence of that state:

$$f_{occurrence}=t_{state}/T\cdot 100\%$$

where *t*_*state*_ is the total occurrence time of the synchronization state and *T* is the total time of LFPs.

All of the signal processing, data analysis, and statistical analysis were performed in MATLAB (Version 7.1 MathWorks Inc., Natick, MA, USA).

## Results

### Wavelet packet basis selection

**Figure 2:**
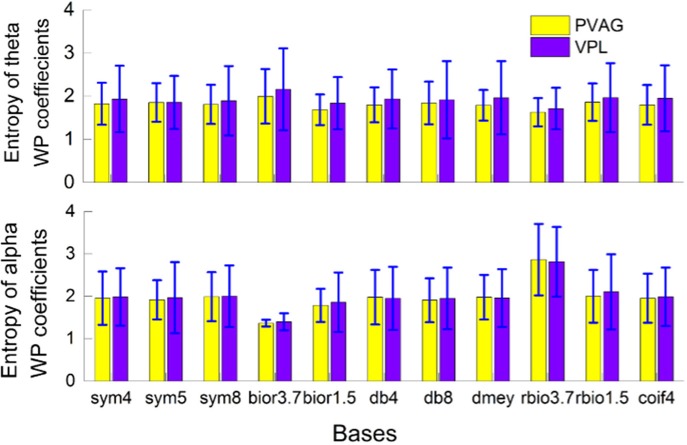
Wavelet packet basis selection based on the entropy of wavelet packet coefficients when characterizing theta and alpha oscillations in the VPL and PVAG LFPs.

A total of 11 WP bases were compared (“sym4,” “sym5,” “sym8,” “bior3.7,” “bior1.5,” “db4,” “db8,” “rbio3.7,” “rbio1.5,” “dmey,” “coif4”). The entropy of WP coefficients of theta and alpha oscillations was computed. As illustrated in Figure 2, there was minimum entropy with the antibiorthogonal wavelet basis “rbio3.7” for the theta band and minimum entropy with the biorthogonal wavelet basis “bior3.7” for the alpha band. This may be because biorthogonal and antibiorthogonal bases are useful for the detection of synchronization oscillations (Supplementary Figure 3). Therefore, in the oscillation extraction model, the “rbio3.7” basis was selected to extract the theta oscillation, and the “bior3.7” basis was selected to extract the alpha oscillation.

**Figure 3:**
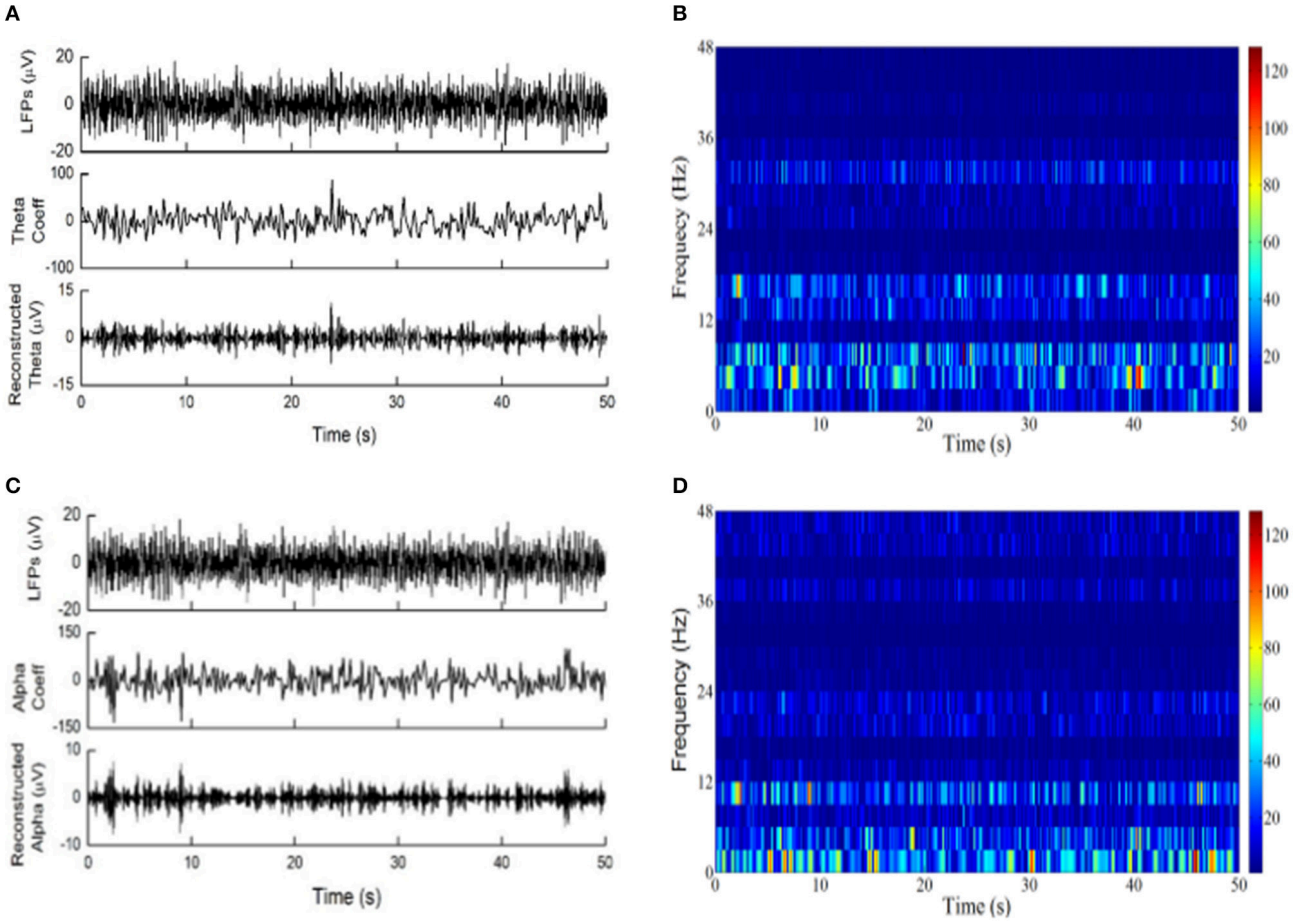
The wavelet packet transform was applied to LFPs. **(A)** From top to down are LFPs recordings of PVAG, the WP coefficients at the theta frequency band with the basis “rbio3.7” and the reconstructed theta oscillation. **(B)** Time-frequency analysis by WP transform with “rbio3.7”; the color bar indicates the absolute value of WP coefficients. **(C)** From top to bottom are the LFPs of PVAG, the wavelet coefficients at the alpha frequency band with the basis “bior3.7” and the reconstructed alpha oscillation. **(D)** Time-frequency analysis by WP transform with “bior3.7”; the color bar indicates the absolute value of WP coefficients.

Examples of LFPs from the PVAG and VPL were WP transformed with “rbio3.7” and “bior3.7.” The theta and alpha oscillations were reconstructed using their respective WP coefficients (Figures 3A,C). The time-frequency spectrogram of LFPs calculated by the WP transform is shown in Figures 3B,D. According to Figure 3, the wavelet basis strongly influences the characterization of the oscillations in LFPs.

### Running window length selection

**Figure 4:**
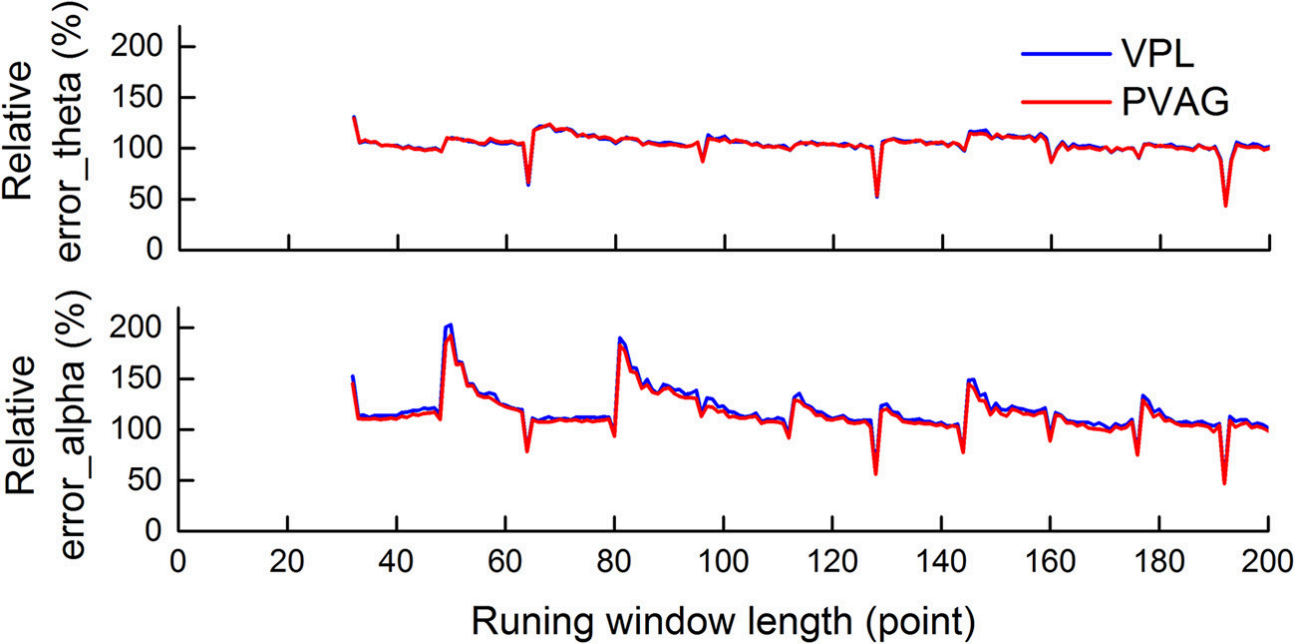
The average relative error over LFP segments was calculated between the reconstructed oscillations with and without the running window. The minimum error was achieved when the window length was a multiple of 64 points. The window length selected was 128.

The length of the running window was selected based on the reconstruction method. Figure 4 shows the average relative error over LFP segments that was calculated between the reconstructed oscillations with and without the running window.

As Figure 4 demonstrates, there were similar results when selecting the running window length in LFPs from both the PVAG and the VPL. For theta oscillations, there was local minimum error when the window length was a multiple of 64 points, i.e., 64, 128, and 192. There was relatively less error at certain positions, i.e., 96 and 160 points. For alpha oscillations, there was also local minimum error when the window length was a multiple of 64 points, i.e., 64, 128, and 192 points. Moreover, the local minimum errors for alpha oscillations were found with more running window lengths than for theta oscillations, e.g., 80, 144 points. The minimum error was achieved when the window length was a multiple of 64 points, which may be related to the level of decomposition. Six-level decomposition was used in this study, and the down-sampling rate of WP decomposition is 64 (2^∧^6).

The window length of 128 points was finally determined for the theta and alpha oscillation extraction by comparing the performance and temporal resolution.

### A priori window length selection

The a priori window length was optimized by comparing the performance of the state identification approach in theta and alpha simulation signals. The sensitivity and specificity were computed when the window lengths were 1, 2, 4, 6, and 8 s. As the a priori window length was increased, the sensitivity of theta and alpha oscillation synchronization state discrimination decreased, and the specificity increased (Table 3). The rate of sensitivity decrease was faster than the rate of specificity increase. Therefore, the length of the a priori window had a greater effect on sensitivity than on specificity. Shorter a priori windows yielded higher sensitivity, while the specificity was near 0.9. Finally, a 2-s window was selected for state discrimination for both the theta and alpha oscillations.

**Table 3:** The performance of synchronization state discrimination for theta and alpha simulation signals using different lengths of a priori windows.

**L_priori_ (s)**	**Theta**		**Alpha**	
	**Sensitivity**	**Specificity**	**Sensitivity**	**Specificity**
1	0.73	0.89	0.64	0.88
2	0.78	0.89	0.66	0.90
4	0.71	0.91	0.54	0.93
6	0.69	0.92	0.50	0.94
8	0.65	0.93	0.45	0.94

*L_priori_ is the length of the a priori window length*.

### Selection of the parameters n1 and n2

There were two parameters in the state discrimination strategy, i.e., *n*_1_ and *n*_2_. For theta oscillations (Table 4), the sensitivity decreased as *n*_1_ increased, while the sensitivity increased as *n*_2_ increased. The sensitivity exceeded 0.8 when *n*_1_ was 1 or when *n*_1_ was 2 and *n*_2_ was larger than 3. The specificity exceeded 0.8 for all combinations. The specificity was ~0.87 when *n*_1_ was 1 or 2. For alpha oscillations (Table 5), a similar trend was found. The sensitivity was 0.8 only for the combination *n*_1_ of 1 and *n*_2_ of 6, and the specificity was near 0.9 for most combinations. Finally, *n*_1_ was set to 1, and *n*_2_ was set to 6 for both theta and alpha oscillation discrimination.

**Table 4:** The performance of the discrimination strategy for theta oscillations with different *n*_1_ and *n*_2_ values.

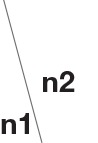	**Sensitivity**						**Specificity**					
	**1**	**2**	**3**	**4**	**5**	**6**	**1**	**2**	**3**	**4**	**5**	**6**
1	**0.82**	**0.85**	**0.87**	**0.89**	**0.91**	**0.93**	**0.91**	**0.88**	**0.87**	**0.87**	**0.86**	**0.86**
2	0.76	0.77	0.79	**0.80**	**0.81**	**0.83**	0.91	0.88	0.88	**0.87**	**0.87**	**0.87**
3	0.75	0.75	0.75	0.78	0.78	0.79	0.93	0.88	0.88	0.88	0.87	0.87
4	0.74	0.75	0.75	0.75	0.77	0.78	0.94	0.90	0.88	0.88	0.87	0.87
5	0.73	0.74	0.74	0.74	0.74	0.77	0.94	0.90	0.89	0.88	0.87	0.87
6	0.72	0.74	0.74	0.74	0.74	0.74	0.96	0.91	0.90	0.89	0.87	0.87

*Bolded values indicate that both the sensitivity and specificity are larger than 0.8*.

**Table 5:** The performance of the discrimination strategy for alpha oscillations with different *n*_1_ and *n*_2_ values.

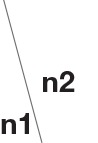	**Sensitivity**						**Specificity**					
	**1**	**2**	**3**	**4**	**5**	**6**	**1**	**2**	**3**	**4**	**5**	**6**
1	0.68	0.72	0.74	0.76	0.78	**0.80**	0.91	0.90	0.89	0.89	0.89	**0.88**
2	0.67	0.68	0.71	0.72	0.75	0.76	0.91	0.90	0.89	0.89	0.89	0.89
3	0.64	0.66	0.66	0.69	0.70	0.72	0.93	0.90	0.90	0.89	0.89	0.89
4	0.62	0.64	0.65	0.65	0.68	0.68	0.93	0.92	0.90	0.89	0.89	0.89
5	0.60	0.62	0.63	0.63	0.63	0.66	0.94	0.92	0.91	0.90	0.89	0.89
6	0.58	0.60	0.61	0.61	0.61	0.61	0.95	0.93	0.92	0.91	0.89	0.89

*Bolded values indicate that both the sensitivity and specificity are larger than 0.8*.

All selected parameters are listed in Table 6 for theta and alpha oscillation synchronization state identification.

**Table 6:** The optimal parameters of the state identification approach for theta and alpha oscillations, respectively.

**Oscillation**	**WP basis**	***L_*running*_***	**L_priori_**	***n_1_***	***n_2_***
Theta	rbio3.7	128 points	2 s	1	6
Alpha	bior3.7	128 points	2 s	1	6

### Method validation

The performance of the approach was further evaluated by 1, investigating the influence of noise on the performance of the state identification approach and 2, testing the advantage of the adaptive thresholding by comparing it with global thresholding.

**Figure 5:**
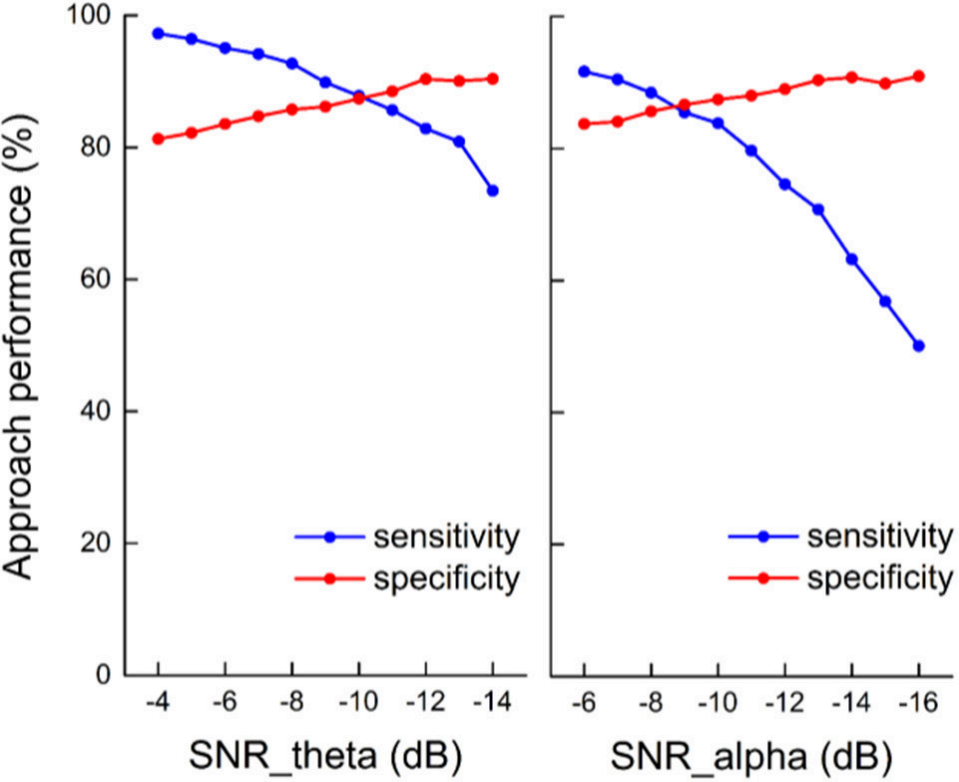
The influence of SNR on the performance of the state identification approach.

**Figure 6:**
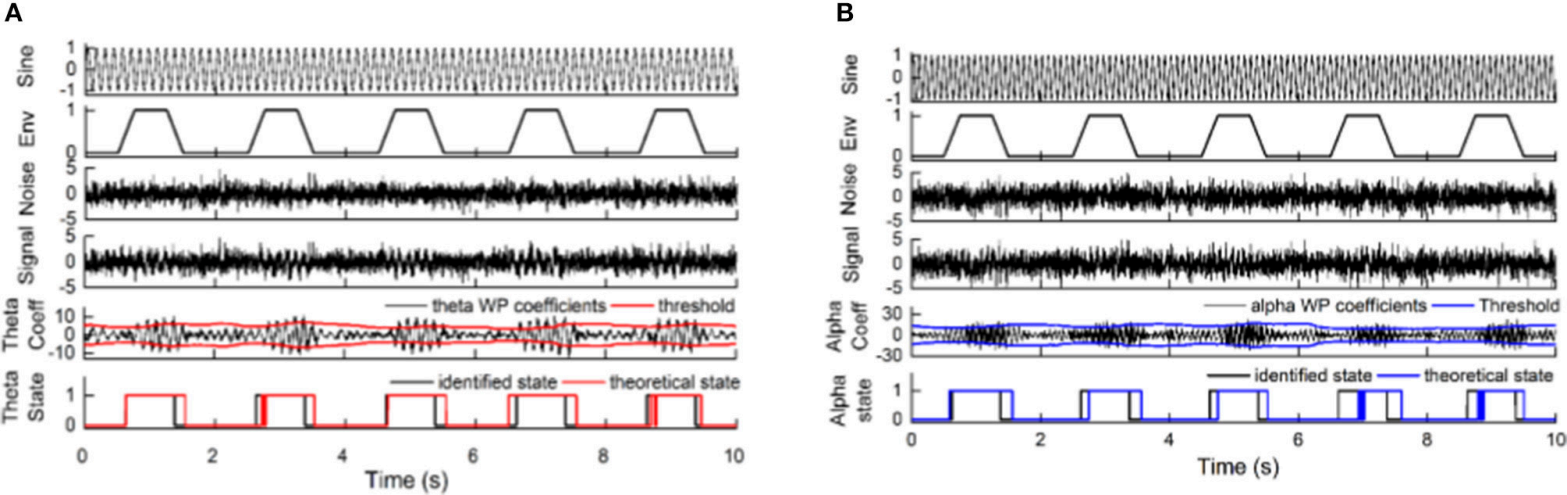
The construction of the simulation signal and the performance of the state identification approach for theta and alpha simulation signals. **(A)** The construction of the theta simulation signal and its synchronization state identified by the state identification approach. **(B)** The construction of the alpha simulation signal and its synchronization state identified by the state identification approach. The pictures, from top to bottom, correspond to the sinusoidal signal (the frequency of the sinusoidal signal is 7.5 Hz for theta simulation and 10.5 for alpha simulation), the trapezoidal envelope, a certain amount of white noise (−9 dB for the theta simulation signal and −11 dB for the alpha simulation signal), the simulation signal by merging the sinusoidal signal, the envelope signal and the noise signal, the coefficients of oscillation and their adaptive threshold, and the performance of the identification compared to the theoretical state of the simulation signal.

The sensitivity and specificity of the state identification approach was computed with an SNR of theta oscillations varying from −4 to −14 dB and an SNR of alpha oscillations varying from −6 to −16 dB in the simulated signals (Figure 5). For theta oscillations, the sensitivity decreased from 98.4 to 78.5%, and the specificity increased from 80.2 to 89.5% as the SNR decreased. For alpha oscillations, the sensitivity was more influenced by the noise, and it markedly decreased as the SNR decreased. The specificity was between 80 and 90% and gradually increased as the SNR decreased. Figure 6 shows the construction of the simulation signal and the performance of the state identification approach for theta and alpha simulation signals. Although the state identification approach identified almost synchronization oscillations, there is still some time delay for identifying.

**Figure 7:**
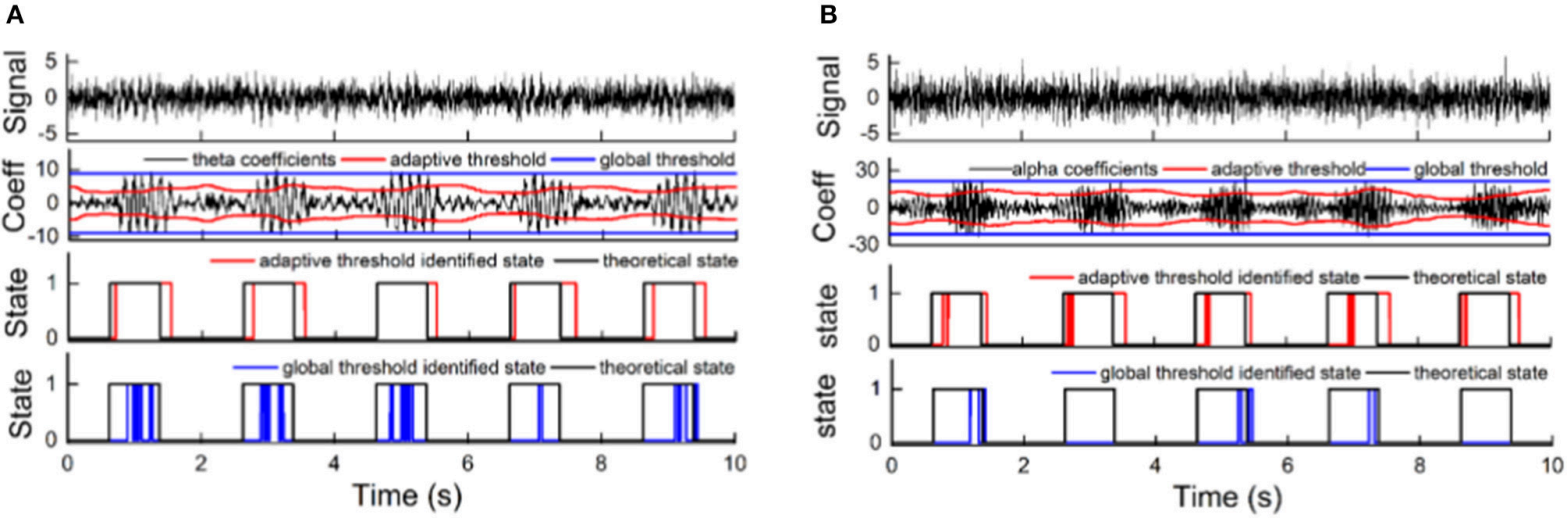
The advantage of adaptive thresholding compared to global thresholding. **(A)** The performance for theta oscillation simulation signal (with −9 dB noise) synchronization state discrimination using an adaptive threshold and a global threshold. **(B)** The performance for alpha oscillation simulation signal (with **–**11 dB noise) synchronization state discrimination using an adaptive threshold and a global threshold. The pictures, from top to bottom, correspond to the simulation signal; the WP coefficients of the oscillation, with its adaptive threshold and global threshold; the synchronization state discrimination performance of the adaptive threshold compared to the theoretical state; and the synchronization state discrimination performance of the global threshold compared to the theoretical state.

The performance of adaptive state identification was compared with that of global state identification with a fixed threshold (Figure 7). A running window of 128 points was used to compute the WP coefficients, and an a priori window of 2 s was used to calculate the adaptive threshold. The fixed threshold was calculated using all WP coefficients of the entire signal and was then used to discriminate the state of each oscillation in the simulated signals. For theta oscillations, the state identification with a fixed threshold achieved 23.7% sensitivity and 97.7% specificity, while the adaptive state identification achieved 87% sensitivity and 87% specificity. For alpha oscillations, the state identification with a fixed threshold achieved 12.5% sensitivity and 98.2% specificity, while the adaptive state identification achieved 79.6% sensitivity and 88.8% specificity. The adaptive strategy greatly improved the sensitivity but compromised the specificity to a certain degree. Moreover, the adaptive strategy increased the stability of the states by reducing the frequent switching between two discriminant states of synchronization and de-synchronization.

### Dynamic neural state identification of LFPs

**Figure 8:**
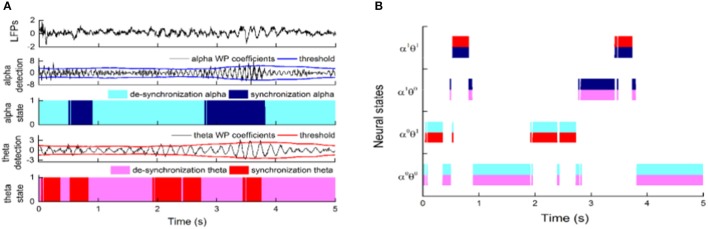
The application of the state identification approach in LFPs and the distribution of neural states based on alpha and theta oscillations. **(A)** The synchronization states of alpha and theta oscillations were discriminated at the same time. From top to bottom, the images correspond to the LFPs; WP coefficients of alpha oscillation and its adaptive threshold; WP coefficients of theta oscillation and its adaptive threshold; and the distribution of the de-synchronization theta oscillation and synchronization theta oscillation over time. **(B)** The distributions of four neural states (α^0^θ^0^, α^0^θ^1^, α^1^θ^0^, and α^1^θ^1^) based on theta and alpha oscillations in the nucleus over time.

**Figure 9:**
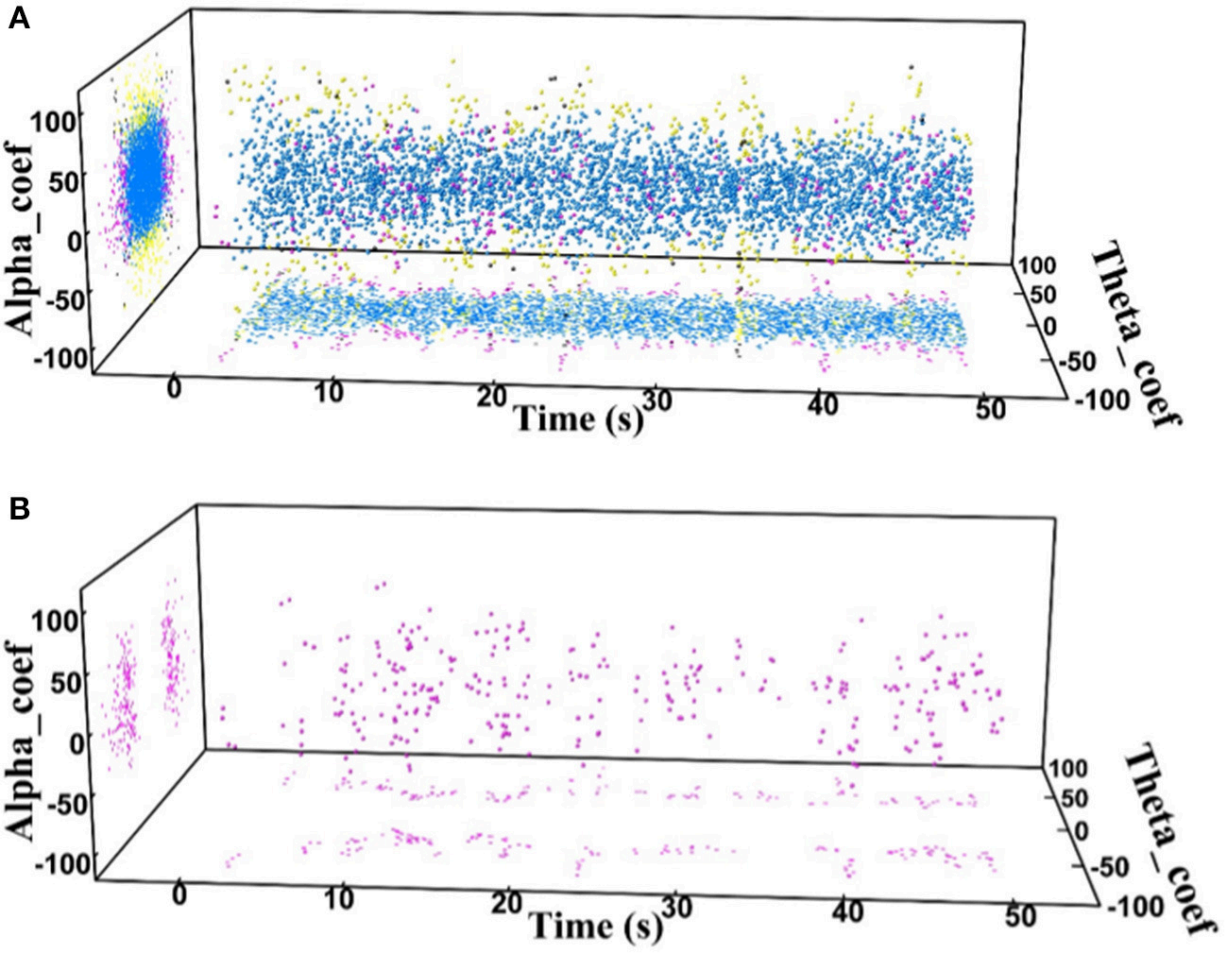
**(A)** The distributions of the four states in one neuropathic pain patient's PVAG over time; α^0^θ^0^ (blue dots), α^0^θ^1^ (pink dots), α^1^θ^0^ (yellow dots), and α^1^θ^1^ (black dots). **(B)** Distribution of the α^0^θ^1^ state over time.

**Figure 10:**
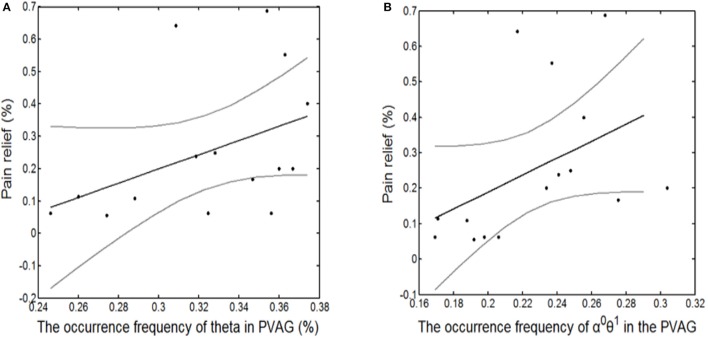
The significantly linear correlation between pain relief and the occurrence timing of the θ^1^ state in the PVAG **(A)** and of the α^0^θ^1^ state in the PVAG **(B)**.

Finally, the state identification approach was applied to encode neuropathic pain by analysis of 27 LFP recordings from the VPL and the PVAG. The WP coefficients of the theta and alpha oscillations were simultaneously extracted by the WP transform, and the adaptive thresholds were calculated for the theta and alpha oscillations (Figure 8A). The synchronization and de-synchronization states of each oscillation were adaptively discriminated. The states for the combination of the theta and alpha oscillations were computed, and four states were identified, i.e., α^0^θ^0^, α^0^θ^1^, α^1^θ^0^, and α^1^θ^1^. The dynamic states are further illustrated in Figure 8B, which shows that the α^0^θ^0^ and α^0^θ^1^ states occurred more frequently. The relationships between the occurrence timing of the six states and pain relief are listed in Table 7. These results illustrate that the occurrence frequency of the α^0^θ^1^ state in the PVAG was significantly positively related to pain relief. However, none of the states in the VPL were significantly related to pain relief. Figures 9, 10 indicate that the correlation between the state α^0^θ^1^ and the pain relief level showed clustering and was more regular than those of other neural states. When the neural state becomes α^0^θ^1^, it will be maintained temporarily, but this does not occur in other neural states. For example, the distribution of the neural state α^0^θ^0^ over time is random.

**Table 7:** The relationships between the average frequency of neural states and pain relief by deep brain stimulation.

**Nucleus**	**Correlation**	**Theta**	**Alpha**	**Alpha-Theta**			
		**θ^1^**	**α^1^**	**α^0^θ^0^**	**α^0^θ^1^**	**α^1^θ^0^**	**α^1^θ^1^**
VPL	r	−0.29	−0.15	0.36	−0.16	−0.23	0
	p	0.33	0.63	0.26	0.61	0.46	1
PVAG	r	0.50	−0.09	−0.44	0.64	−0.19	0.01
	p	0.05^*^	0.74	0.11	0.01^**^	0.49	0.96

The values were calculated as the Spearman correlation (

* *p < 0.05*,

** *p < 0.01)*.

## Discussion

In the VPL and the PVAG nuclei, the theta, alpha, beta, and gamma neural oscillations of LFPs are associated with neuropathic pain. These oscillations can be characterized by measures of amplitude, spectra, and other parameters, which have been used to investigate the association of neural activities with pain and pain relief. One study used alpha and beta oscillation power to automatically classify pain levels (Zhang et al., 2013).

Neurophysiological or pathological processes are simultaneously related to time-varying neural oscillations. There is a demand for the development of a framework to integrate multiple oscillations to construct neural states in high dimensions. We explored the features of multiple oscillations correlated with neuropathic pain in three dimensions (Huang et al., 2016b). In this study, we further enriched the framework by developing approaches to identify the dynamic neural states of multiple oscillations with sparse representation and adaptive discrimination. Such an approach is useful for neural coding, neurophysiological mechanistic research in neuropathic pain, and other neurological diseases, such as Parkinson's disease, epilepsy (Cotic et al., 2011; Wu et al., 2013), and other psychiatric diseases (Neumann et al., 2014). Ultimately, it could be valuable for the development of a neural state-dependent adaptive DBS system.

In the state identification approach, the windowed WP transform provides more flexible decomposition to extract multiple neural oscillations, and its simplicity makes it easier to implement in real time (Li et al., 2016), which will be valuable for the development of closed-loop deep brain stimulation (Priori et al., 2012). The inter-related parameters of the feature extraction model and the state discrimination model were systematically optimized to improve the neural state identification in terms of sensitivity and specificity. The key parameters were the WP basis and the running window length for specific neural oscillations in the oscillation extraction model, and the key parameters were a priori window length and discriminant strategy for the discrimination model. The state identification approach was evaluated with simulated signals. The functional relevance of neural states was investigated by correlating the measures of the neural states with pain relief by DBS. The performance of state identification was improved with systematic optimization. First, the sparse representation method of the WP transform was chosen to represent the activity of oscillations, as it provides a more flexible frequency division to meet the frequency band shift of neural oscillations. In this paper, it has been found that “bior3.7” worked significantly better than other bases in alpha oscillations of LFPs. This may be due to its specific shape, which is similar to the neural oscillations. More importantly, it demonstrates that the sparse representation with a specific basis could be able to significantly enhance the representation of certain neural oscillations. Bases can be trained from the LFPs of individual subjects with lifting (Subasi and Erçelebi, 2005), and the sparse representation with such an individualized basis could be a more efficient coding strategy. The WP transform has been well-developed, and its simplicity makes it possible to implement in real time (Zandi et al., 2010). By transforming the neural oscillations from the time domain to the wavelet domain, the random and irregular signals were represented by evenly distributed coefficients with low values, while the oscillations and regular signals were represented by sparsely distributed WP coefficients with high values (Donoho and Johnstone, 1994). The synchronization oscillations became a series of high-value WP coefficients, in contrast to other de-synchronization oscillations with low values. The WP transform enhances the representation of the synchronization oscillations and therefore highlights the contrast with de-synchronization oscillations.

Moreover, the oscillation extraction model was developed to capture the synchronization neural oscillations with the enhanced sparse representation in the wavelet domain. The performance of the oscillation extraction model was improved by optimizing the wavelet basis and the length of the running window for specific oscillations. The proper running window balanced the timing resolution and accuracy of the identification performance.

Wavelet packet transform is one method used to achieve sparse representation. The significance of sparse representations is that a small amount of signal can be used to represent a large amount of data. Sparse representations have been widely used in information coding (Cocchi et al., 2001), data compression (Walczak and Massart, 1997; Meyer et al., 1998), and de-noising (Tikkanen, 1999; Fathi and Naghsh-Nilchi, 2012). Previous studies have demonstrated that neural activities exhibit transient, spindle-shaped dynamic oscillatory behaviors. These activities of interest usually exhibit more regular patterns than background activity, and therefore, sparse representation is able to efficiently characterize these neural oscillations. For neural state identification, the sparse representation should be combined with a discrimination model. The WP transform provides a solution for both (Hou and Shi, 2016), and the computation cost is lower than for dictionary-based methods (Chang et al., 2016). In the future, improved sparse representation approaches, such as shapelet-based sparse representation (Roscher and Waske, 2016), global regularization (Shu et al., 2016), or match pursuit (Cui and Prasad, 2016), could be combined with machine learning approaches, such as Bayesian approaches (Liu, 2016).

Second, in the state discrimination model, three aspects were incorporated to improve the discrimination performance: a thresholding strategy, an a priori window, and a customized discrimination strategy. Time-varying thresholding ensured that the discrimination model was adaptive to dynamically changing oscillations. The a priori window balanced robustness and time resolution and provided a robust threshold prediction for future data. The customized discrimination strategy based on a *post-hoc* test further improved the performance by reducing the occasional influence with optimized selection of the *n*_1_ and *n*_2_ values. Such a posteriori discrimination provided a compromise between sensitivity and specificity, and this improved the specificity and made the discrimination model robust against noise. Although the a posteriori discrimination induced a time delay for state identification, it could be compensated for by re-aligning the on/off timing of the states for off-line applications. The mean time delays for identifying the synchronization state were found to be 45 ± 54 and 109 ± 88 ms for theta and alpha simulation signals, respectively. For de-synchronization states, the mean time delay was 165 ± 59 and 158 ±76 ms for theta and alpha simulation signals, respectively. Nevertheless, it was still short enough for adaptive deep brain stimulation in real time. In the future, the Bayesian method could further improve the discrimination performance by statistically incorporating a priori and a posteriori information.

Pain is an integrative phenomenon that results from dynamic interactions between sensory and contextual processing (Melzack and Casey, 1968). Pain processing may be related to complex neural states. In this study, pain relief was correlated to the neural states of theta and alpha oscillations with binary encoding. Pain relief was not only related to the synchronization timing of theta oscillations but was also specifically related to the α^0^θ^1^ state.

In the current study, the amplitude was used as a synchronization feature for neural state identification. However, the neural states may also be related to other types of features. Phase synchronization and coupling between multiple oscillations were also found to be associated with pain (Sarnthein and Jeanmonod, 2008). Patterns of neural oscillations have also been measured with Lempel-Ziv complexity (Geng et al., 2017), coefficients of variance (Little et al., 2012), and entropy (Darbin et al., 2016). When features in multiple domains are used to encode neural states, the binary coding method may become no longer intuitive or applicable, and non-linear coding with Bayesian and artificial neural network methods would be needed.

The state identification approach could be generalized for neural state identification in other neurological diseases. The approach in this study dealt with a more challenging situation in that the PVAG and VPL LFPs have higher background random neural activities than the subthalamic LFPs in Parkinson's disease. Previous research has shown that beta oscillations in subthalamic LFPs exhibit more regular patterns (Ray et al., 2008) resulting from resonant subthalamo-cortical circuits (Eusebio et al., 2009). Our preliminary research has shown that beta oscillations can be reliably identified using the state identification approach (Zhang et al., 2017), which will provide an essential step toward closed-loop stimulation adaptive to the rhythms of neural activities.

The state identification approach may be valuable for the development of closed-loop DBS in the future. The approach provides a method to capture multiple oscillations comparable to online processing. The state identification approach may be more robust than the solely amplitude-based methods (Little et al., 2013) in situations with high level noise because the patterning oscillations are enhanced, and the thresholding is adaptive with the change of oscillations. Moreover, the approach is flexible in adjusting the sensitivity and specificity, which would be valuable for balancing the efficacy and side effects of the treatment. The higher sensitivity may lead to better relief of the symptoms by more accurately modulating the neural oscillations of interests, while the higher specificity may reduce the potential side effects with less stimulation at states irrelevant to the pathological origin. However, there are some potential limitations to translating the state identification approach into closed-loop DBS. The main potential limitation will be the quality of LFPs for long-term recording. Both the impedance of the implanted electrode and stimulation artifacts will influence the quality of LFPs. However, the impedance of implanted electrodes varies among different studies and patients (Abosch et al., 2012; Lungu et al., 2014; Satzer et al., 2014). Researchers have found that the influence of impedance is not significant (Steiner et al., 2017). The more challenging issue is stimulation artifacts, especially those arising from low frequency stimulation. When 20 Hz stimulation is used in neuropathic pain, preprocessing of a low pass filter should be applied, and the filter needs to be purposely designed, and independent component analysis may be useful to further reduce stimulation artifacts. Alternatively, recording from cortical or subcortical electrodes may be used to develop closed-loop deep brain stimulation (Rosin et al., 2011).

In summary, this study provides a robust approach to identifying the dynamic neural states of deep brain nuclei. In the future, this work may advance closed-loop deep brain stimulation based on neural states integrating multiple neural oscillations.

## Ethics statement

The study was carried out in accordance with the Declaration of Helsinki and received approval from the Oxford Research Ethics Committee B (project number 13 SC 0298).

## Author contributions

HL: Design of the study, analysis and interpretation of the data, drafting and revising the manuscript; YH: Design of the study, analysis and interpretation of the data; XD: Analysis and interpretation of the data; YZ: Data collection; AG: Clinical support, data collection, interpretation of the data; TA: Clinical support, data collection; SW: Design and conceptualization of the study, conduction of the experiment, analysis and interpretation of the data, drafting and revising the manuscript critically for important intellectual content.

### Conflict of interest statement

The authors declare that the research was conducted in the absence of any commercial or financial relationships that could be construed as a potential conflict of interest.
