# Supplementary material for: Dynamic Neural State Identification in Deep Brain Local Field Potentials of Neuropathic Pain
Source: Front Neurosci. 2018 Apr 11;12:237. doi: 10.3389/fnins.2018.00237 (PMC5904287; doi:10.3389/fnins.2018.00237)
Supplement: Supplementary file 1 [file Presentation1.PDF]

Supplementary Material

Dynamic neural state identification in deep brain local field potentials of neuropathic pain

Huichun Luo, Yongzhi Huang, Xueying Du, Yunpeng Zhang, Alexander L. Green, Tipu Z. Aziz, Shouyan Wang*

*** Correspondence:** Shouyan Wang: shouyan@fudan.edu.cn

1 Wavelet packet transform

1.1 Discrete wavelet transform

A discrete wavelet transform algorithm usually consists of two stages of decomposition and reconstruction (Mallat, 1989;Rioul and Vetterli, 1991;Daubechies, 1992;Samar et al., 1999;Walden, 2001). The diagram of multi-level decomposition is illustrated in **Supplementary Figure 3**. In the first decomposition level, the original signal x_k (k =0,…,N-1) is decomposed into low-frequency and high-frequency components: the former s_k^1 (approximate coefficients) are obtained by low-pass filtering x_k by scaling filter g_l (l =0,…,L-1) and downsampling by two; similarly, the latter d_k^1 (detail coefficients) can be obtained by high-pass filtering x_k by wavelet filter h_l (l=0,…,L-1) and downsampling by two. The next level of decomposition can then be computed using the approximate coefficients of the previous level as the new input. The detail coefficients d_k^j and the approximate coefficients s_k^j are the jth level decomposition output in the DWT pyramid algorithm as following:

$$d_k^j = \sum_{l=0}^{L-1} h_l s_{(2k+1-l) \bmod N_{j-1}}^{j-1},$$

$$s_k^j = \sum_{l=0}^{L-1} g_l s_{(2k+1-l) \bmod N_{j-1}}^{j-1}, \quad N_j = \frac{N}{2^j}$$

in which, the filters h_l and g_l can be derived from different wavelet bases. The reconstruction stage of the algorithm is the inverse wavelet transform. The decomposed detail and approximate coefficients are up-sampled by two and then the up-sampled signals are convolved with the reconstruction filters $h_l^{'}$ and $g_l^{'}$ to reconstruct the approximate coefficients as the input of next level reconstruction.

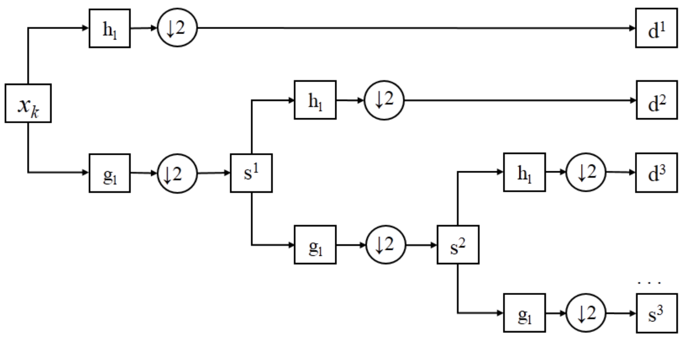

Supplementary Figure 1. The diagram of multi-level decomposition. It is decomposed into detail coefficients $d^{(1,2,3,\ldots)}$ (high frequency component) and approximate coefficients $s^{(1,2,3,\ldots)}$ (low frequency component) by discrete wavelet transform.

1.2 Wavelet packet transform

The wavelet packet (WP) transform is a generalization of the DWT, in which the decomposition procedure is done in both lower and higher frequencies. This general decomposition offers a greater range of possibilities for signal analysis than the discrete wavelet decomposition (Percival and Walden. 2006). In the WP tree (**Supplementary Figure 4**), node [a, b] is the *b*th node at decomposition level from the root of the WP tree, for b=0, 1, ..., L, and a =0, 1,... , 2 −1, where L = $\log_2$ n (n is the signal dimensionality). The 'frequency resolution' at each level of the tree is defined as the number of nodes associated with the band-width of 1 Hz. In each decomposition step, the frequency band associated with each node (parent) is divided evenly into two subbands though a low-pass filter and a high-pass filter which come from wavelet basis. Therefore, at the *a*th decomposition level, each node is associated with bandwidth of Fs/2+1, where Fs is the sampling frequency.

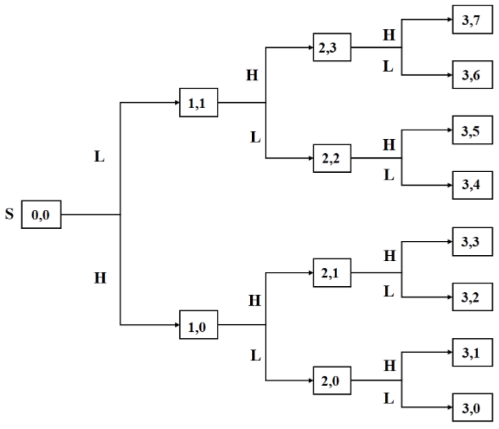

Supplementary Figure 2. Decomposition diagram of three levels wavelet packet. The input signal (S) was decomposed into 3 level. In each decomposition step, one node was decomposed by a low-pass filter (L) and a high-pass filter (L).

1.3 Wavelet basis

There were 11 bases chose as candidates for basis optimization. According to their shape (**Supplementary Figure 5**), the ‘bior3.7’ is more similar to spindle-shaped oscillation.

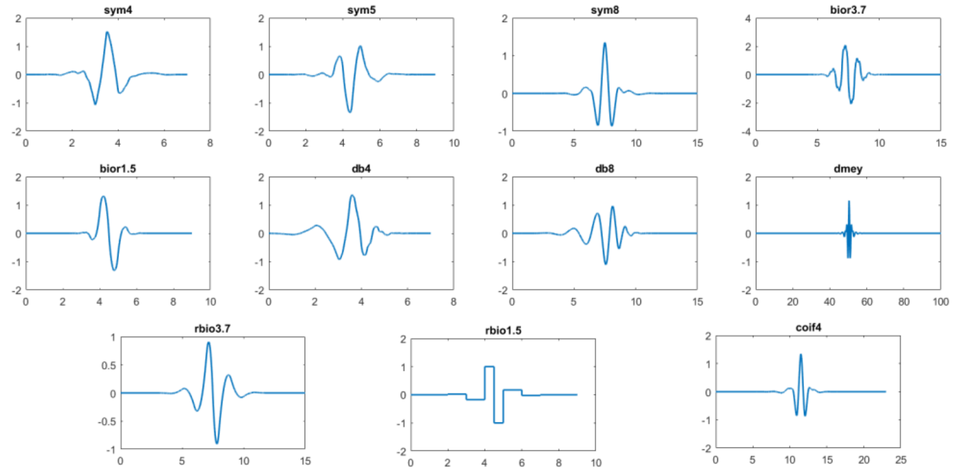

Supplementary Figure 3. The shape of wavelet bases. There were total 11 basis compared: ‘sym4’, ‘sym5’, ‘sym8’, ‘bior3.7’, ‘bior1.5’, ‘db4’, ‘db8’, ‘rbio3.7’, ‘rbio1.5’, ‘dmey’, ‘coif4’.

2 Matlab code of the state identification approach

Matlab code of the state identification approach. The code includes three functions: ‘main’, ‘SIA’, ‘med_num’ function.

```
%main function
%load data at first
%the matrix NS is the neural state of signal
nn1=1;nn2=6;nn3=1;nn4=6;
data=resample(signal,384,500);   %resample to 384Hz
[C_theta,CstimOut_theta,Cthre_theta,t]=SIA(data,'rbio3.7',[6 3],nn1,nn2);
[C_alpha,CstimOut_alpha,Cthre_alpha,t]=SIA(data,'bior3.7',[6 2],nn3,nn4);
% decoding neural state
    for j=1:length(CstimOut_theta)
        a=S_theta(1,j)+S_alpha(1,j);
       if (a==2)
          NS(1,j)=4;
       else
           if (a==0)
              NS(1,j)=1;
           else
               if (S_theta(1,j)==1)
                NS(1,j)=2;
               else
                 NS(1,j)=3;
               end
           end
       end
    end
```

```
function [WPT_coef,StimOut,Thre,t]=SIA(signal,basis,node,n1,n2)
%the function for achieve state identification approach in matlab
%the size of the input signal was 1xN

% signal=resample(alpha,384,500);
%  signal=alpha;
%  wptpara.cnode=[6 2];
%  wptpara.wf='bior3.7';
%  n1=1;n2=6;

%clear all; clc;
F=384;          % sampling rate
dwtmode('sym');
wptpara.wf=basis;    % the wavelet packet decomposition parameters
wptpara.ent='shannon';    % the entropy
wptpara.lev=6;      % decomposition level
wptpara.cnode=node;    % the node to extract beta component
wptpara.thsel='minimaxi';
Dwinstep=round(0.02*F);    % the step to move
Dwinlen=128;
Twinlen=round(2.0*F);     % the window length to calculate threshold
N=2^wptpara.lev;
n=ceil(Dwinlen/N);


%------------------------ initialization------------------------%
StimOut=zeros(size(signal));
StimOut2=[];
Coef=[];     %priori WP coefficients
WPT_coef=[];    %WP coefficients
Thre=zeros(1,length(signal));   %threshold
c=1;
stp=1;
edp=Dwinlen;
%initiate the priori window
while edp<=Twinlen
  data=signal(stp:edp);
  WPTree=wpdec(data,wptpara.lev,wptpara.wf,wptpara.ent);
  dwptc=wpcoef(WPTree,wptpara.cnode);
  dwptc=med_num(dwptc,n);    %Remove redundant WP coefficients
  Coef=[Coef,dwptc];
  c=c+1;
  stp=stp+Dwinstep;
  edp=edp+Dwinstep;
end
num1=length(dwptc);
```

```
coefn=length(Coef);
WPT_coef=[WPT_coef,Coef];
lfpn=length(signal);
Window_state=zeros(1,num1);   %current synchronization state
StimOut2=[StimOut2, zeros(size(Coef))];
Th=zeros(size(StimOut2));

%-------------------------identification start ----------------%
mark1=zeros(1,n1+1);
mark2=zeros(1,n2+1);
state=0;
ss=zeros(1,n);
while edp<=lfpn
  m=median(abs(Coef))/0.6745;
  th=thselect(Coef./m,wptpara.thsel)*m;
  Thre(1, stp:edp)=th;
  data=signal(stp:edp);
  WPTree=wpdec(data,wptpara.lev,wptpara.wf,wptpara.ent);
  dwptc=wpcoef(WPTree,wptpara.cnode);
  dwptc=med_num(dwptc,n);
     if length(find(abs(dwptc)>th))>0
          temp1=1;
     else
         temp1=0;
     end
     mark1(1,1:n1)=mark1(1,2:end);
     mark1(1,n1+1)=temp1;
     mark2(1,1:n2)=mark2(1,2:end);
     mark2(1,n2+1)=temp1;
     if abs(mark1(1,1)-1)<0.0001
         if sum(mark1)>n1
             StimOut(1,edp-(n1+1)*Dwinstep+1:edp-n1*Dwinstep)=1;
             ss(:,:)=1;
             state=1;
         else
             StimOut(1,edp-(n1+1)*Dwinstep+1:edp-n1*Dwinstep)=state;
             ss(:,:)=state;
         end
     end
     if abs(mark2(1,1)-0)<0.0001
          if sum(mark2)==0
             StimOut(1,edp-(n2+1)*Dwinstep+1:edp-n2*Dwinstep)=0;
             ss(:,:)=0;
             state=0;
         else
             StimOut(1,edp-(n2+1)*Dwinstep+1:edp-n2*Dwinstep)=state;
             ss(:,:)=state;
         end
```

```
    end
  Coef(1:coefn-num1)=Coef(num1+1:coefn);
  Coef(1,coefn-num1+1:coefn)=dwptc(1,:);
  WPT_coef=[WPT_coef,dwptc];
  StimOut2=[StimOut2,ss];
  Window_state(:,:)=state;
  Th=[Th,th]; Th=[Th,th];
  c=c+1;
  stp=stp+Dwinstep;
  edp=edp+Dwinstep;
end
t=(Dwinlen/2-Dwinstep/4)/F:Dwinstep/2/F:(edp-Dwinlen/2-3*Dwinstep/4)/F;
t1=1/F:1/F:length(signal)/F;

%----------------------draw picture---------------------------------%
% figure; subplot(211);plot(t1,signal); title('the raw data');
% subplot(212);plot(t,WPT_coef,'b',t1,Thre,'r',t1,-Thre,'r', t1,StimOut,'b');title('the coefficient of alpha rhythm');




function A=med_num(data,N)
% this function  from the 1xM matrix  to select the middle N numbers

L=length(data);
if mod(L,2)==1
    if mod(N,2)==1
        A=data((L+1)/2-(N-1)/2:(L+1)/2+(N-1)/2);
    else
        A=data((L+1)/2-N/2:(L+1)/2+(N-2)/2);  %one more for right
    end
else
    n=L/2;
    if mod(N,2)==1
        n1=n;n2=n1+1;
        if data(n1)>data(n2);       %chose the bigger one
            A=data(n1-(N-1)/2:n1+(N-1)/2);
        else
            A=data(n2-(N-1)/2:n2+(N-1)/2);
        end
    else
        A=data(L/2-(N-2)/2:(L+2)/2+(N-2)/2);
    end
end
```

Reference：

Green A.L., Wang S., Stein J.F., Pereira E.A.C., Kringebach M.L., Liu X., et al. (2009). Neural signatures in patients with neuropathic pain Neurology. 72(6) 569-71. doi: 10.1212/01.wnl.0000342122.25498.8b

Percival, D.B and Walden, A.T. (2006). Wavelet methods for time series analysis (Vol. 4). Cambridge university press.

Zhang, K., Feng, B., Zhang, Y.P., Huang, Y.Z., and Wang, S.Y. (2017). Pattern Identification of Subthalamic Local Field Potentials in Parkinson’s Disease. In ITM Web of Conferences (Vol. 12, p. 02004). EDP Sciences. doi:10.1051/itmconf/20172004.

Zhang K., Du X.Y., Huang Y.Z., Luo H.C and Wang S.Y. (2017). Feature Detection of Subthalamic Local Field Potentials in Parkinson’s Disease. Space Medicine & Medical Engineering. doi:10.16289/j.cnki.1002-0837.2017.03.010
